# Effects of water–nitrogen coupling on water and salt environment and root distribution in Suaeda salsa

**DOI:** 10.3389/fpls.2024.1342725

**Published:** 2024-02-19

**Authors:** Qiang Xu, Hongguang Liu, Mingsi Li, Gong Ping, Pengfei Li, Yibin Xu, Qian Zhang, Hanji Xia

**Affiliations:** ^1^ College of Water Conservancy & Architectural Engineering, Shihezi University, Shihezi, China; ^2^ Key Laboratory of Modern Water-Saving Irrigation of Xinjiang Production & Construction Group, Shihezi, China

**Keywords:** Suaeda salsa, saline wasteland, root distribution, drip irrigation, root zone

## Abstract

Understanding the spatial distribution of crop roots is crucial for effectively managing crop water and fertilizer. We investigate the effects of water–nitrogen coupling on the water–salt environment and root distribution in the root zone of *S. salsa*. Three irrigation levels were established, calculated according to 0.35 (W1), 0.50 (W2), and 0.65 (W3) of local *ET_0_*. The three nitrogen levels were 150 (N1), 250 (N2), and 350 (N3) kg·hm^−2^ in a complete combination design. With the augmentation of irrigation water and nitrogen application, the total root weight density of the root system of Suaeda salsa increased from 17.18×10^-3^ g·cm^-3^ to 27.91×10^-3^ g·cm^-3^. The distribution of soil water suction significantly influences the root distribution of Suaeda salsa in saline soil, causing a transition from a narrow deep type to a wide shallow type. Under the W2 irrigation level, soil water suction ranges from 1485.60 to 1726.59 KPa, which can provide water for *S. salsa*.it becomes feasible to attain the necessary water and salt environment for the growth and development of *S. salsa*, resulting in the attainment of maximum biomass, ash content, and salt uptake. No significant differences in the biomass, ash content, and salt uptake of *S. salsa* was noted between N2 and N3 nitrogen application levels (*p* > 0.05).The optimal irrigation volume and nitrogen application level were 0.50 *ET_0_* and 250 kg·hm^−2^, respectively. The results of this study provide a scientific basis for the large-scale planting of *S. salsa* in extreme arid areas to improve and utilize saline wastelands.

## 1 Introduction

Salinized soil is widely distributed worldwide, and the problem of soil salinization is a global challenge (Chang et al., 2023). In China, saline–alkali land covers an area of approximately 1 × 10^8^ hm^2^, accounting for about 10% of the world’s total saline–alkali land area (Thomas et al., 2013; Amini et al., 2016). Xinjiang is the largest saline–alkali soil area in China, spanning 2.2 × 10^7^ hm^2^, accounting for 36.80% of the country’s saline–alkali soil land (Li et al., 2016). These lands are viewed as strategic reserves for arable land, offering substantial potential for agricultural development (Luo, 2012). The rational development and utilization of saline–alkali land is a topic of increasing interest. *Suaeda salsa* play a key role in this context. These plants can actively absorb salt from the soil and accumulate it in their parts above the ground. Harvesting these parts can help improve the quality of saline–alkali land (Liang and Shi, 2021). Moreover, *S. salsa* can be used as livestock feed (Wang et al., 2022). Therefore, the utilization of *S. salsa* as feed can drive the large-scale development and use of saline–alkali lands (Zhang et al., 2005, 2018).

The root system is crucial for crops because it absorbs nutrients and water, substantially affecting crop growth, development, and yield (Yan, 2016; Li et al., 2019). Roots exhibit a hydrophilic distribution in soil (Hu et al., 2017). He (2021) revealed that soil matrix suction, instead of soil moisture content, predominantly affects the distribution of roots. This is because root cells require soil water for growth, and a low soil matrix potential can hinder root growth even if the moisture content is high (Li et al., 2019). Li (2006) reported that water deficit can increase the depth of root penetration, biomass of deeper roots, and horizontal distribution of roots. In addition, Liang et al. (2015) determined that under salt stress, crops tend to increase their root cap ratio to meet water requirements.

The nutrient status substantially affects the growth and distribution of roots (Guo et al., 2022). In areas with uneven salt distribution, the absorption of water and nutrients by roots decreases in high-salt areas but markedly increases in low-salt areas, demonstrating a compensatory effect of roots on the absorption of water and nutrients (Feng et al., 2011). Li et al. (2015b) investigated the effect of different nitrogen levels on the root architecture of cotton. Under low nitrogen conditions, crops improve their nitrogen supply by increasing root volume and the root-to-shoot ratio. By contrast, under high nitrogen conditions, root growth is often inhibited once the nitrogen accumulation in the plant reaches a certain threshold.

Shahnazari et al. (2008) found that there is still a significant coupling effect between water and fertilizer. Under mild water deficit, increasing nitrogen application can significantly increase the dry weight and volume of tomato roots, promote root penetration, and show a trend of shallow rooting in the high nitrogen treatment. Yu et al. (2022) believed that in a certain range of water and fertilizer, the dry weight of cotton roots under drip irrigation under plastic film increased with the increase of fertilizer amount, but the yield tended to decline after exceeding a certain threshold. Suitable water and fertilizer conditions can promote root growth. Xie and Tian (2010) found that nitrogen application was too high, increasing irrigation did not alleviate the inhibitory effect of excessive nitrogen on root dry mass density (Li et al., 2019).

In addition, under non-uniform distribution of salt, the absorption of water and nutrients by roots in high salinity areas will decrease. In low salinity areas, the absorption of roots significantly increases, indicating a compensatory effect of water and nutrient absorption by roots (He, 2021). In *S. salsa* root zone located in high salt environment, it is of great significance to study the influence of water-nitrogen coupling on water and salt environment and root distribution, and to clarify the spatial distribution of crop roots. However, studies on the growth and distribution of *S. salsa* roots in saline wastelands under drip irrigation are limited. Therefore, this study 1) investigated the regulatory effect of water–nitrogen coupling on the water–salt environment in saline wastelands, 2) examined changes in *S. salsa* root growth and spatial distribution in the water–salt environment, and 3) evaluated the effect of water–nitrogen coupling on the structure and salt uptake capacity of *S. salsa*. The findings of this study can provide a theoretical basis for optimizing water–nitrogen application in cultivating *S. salsa*, thereby improving the water–salt environment in saline wastelands.

## 2 Materials and methods

### 2.1 Overview of test sites

**Figure 1:**
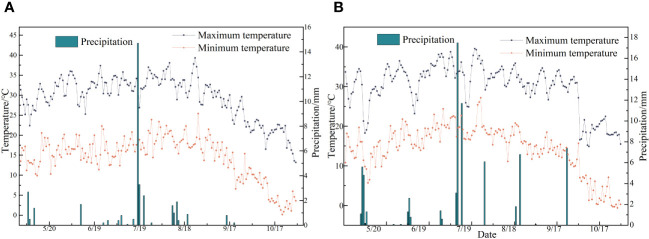
**(A, B)** Represent the temperature and precipitation data of experimental points from May to October in 2022 and 2023, respectively.

This study was conducted from April 2022 to September 2023 at the Modern Agricultural Industrial Park in Bachu County, Kashgar Prefecture (78°55′E, 39°75′N, elevation 1072.80 m). Situated in the southwest of the Xinjiang Uygur Autonomous Region, Bachu County lies at the southern base of the Tianshan Mountains and the northwest edge of the Tarim Basin. The area experiences a temperate continental arid climate characterized by scarce rainfall and drought conditions. The average annual temperature is 11.8°C, with an annual sunshine of 4434 hours. The annual frost-free period is 213 days, the average annual precipitation is approximately 50 mm, and the average annual evaporation is over 2500 mm (refer to meteorological data in **Figure 1**). The physical properties of the topsoil (0–40 cm depth) at the field site are listed in **Table 1**.

**Table 1:** Physical and chemical properties of soil.

Soil propertices	Soil depth/cm			
	0~10	10~20	20~30	30~40
Sand/(%)	15.86	18.36	13.52	16.52
Silt/(%)	79.90	75.96	72.52	73.26
Clay/(%)	4.24	5.68	13.96	10.22
Bulk density/(g·cm^-3^)	1.68	1.70	1.69	1.72
Field capacity/(cm^3^·cm^-3^)	19.86	20.65	20.26	19.52
SOM/g·kg^-1^	2.63	1.96	1.35	1.52
Total N/(g·kg^-1^)	0.26	0.21	0.16	0.12
Total P/(g·kg^-1^)	0.31	0.25	0.21	0.14
Total K/(g·kg^-1^)	10.25	7.32	5.62	6.02
Available N/(mg·kg^-1^)	8.26	7.62	8.23	6.23
Available P/(mg·kg^-1^)	6.52	5.22	3.52	4.52
Available K/(mg·kg^-1^)	103.08	62.63	51.23	50.58
Salt content/(g·kg^-1^)	142.51	56.68	37.23	28.64
pH	8.2	8.1	8.3	7.8

### 2.2 Experimental design

This experiment was based on meteorological data from Bachu County Meteorological Station in Kashgar region from April to September during 2020 to 2022.We used the FAO-56 Penman Monteith (FPM) formula, as recommended by the Food and Agriculture Organization, to calculate the reference evapotranspiration (*ET_0_*). Because of the absence of the crop coefficient (*K_c_*) of *S. salsa*, the irrigation quota was directly calculated based on the local *ET_0_* values of 0.35, 0.50, and 0.65, denoted as W1, W2, and W3, respectively. The nitrogen application rates were set at 150, 250, and 350 kg·hm^−2^ (Pan, 2014), denoted as N1, N2, and N3. Each plot spanned an area of 50 m^2^, with 3 replicates per treatment. To prevent the lateral movement of water and fertilizer, 70 cm-wide ridges were constructed between plots. *S. salsa* seeds were collected from the Halophyte Garden at the Fukang Desert Ecological Experimental Station of the Xinjiang Institute of Ecology and Geography, Chinese Academy of Sciences. Before sowing, the experimental area was leveled. Artificial trenching and strip sowing methods were used to sow *S. salsa* on both sides of the drip irrigation belt within a 15 cm range. The plant and row spacing were set at 50 cm and 30 cm, respectively, with a seeding rate of 30 kg·hm^−2^. Drip irrigation was employed, using water from the Yeerqiang River with a mineralization degree of 1.24–1.58 g·L^−1^. Seeds were sown on April 25, 2022, and April 27, 2023, using a “dry sowing and wet discharge” method. Seedlings were established by May 20, with a density of 40 plants·m^−2^. The irrigation and nitrogen application rates for each treatment are listed in **Table 2**. Phosphorus and potassium were used as base fertilizers at rates of 150 kg·hm^−2^ of P_2_O_5_ and kg·hm^−2^ of K_2_O 75, respectively (see **Table 2**).

**Table 2:** Division of *Suaeda salsa* growth period and irrigation water amount.

Number of irrigation	Irrigation date	Days after sowing	Irrigation water volume/(mm)			Nitrogen fertilizer application rate/(kg·hm^-2^)		
2022			W1	W2	W3	N1	N2	N3
1	4.25	1	23	28	37	0	0	0
2	5.2	8	20	29	36	15	25	35
3	5.8	14	20	32	37	20	25	40
4	5.16	22	24	30	38	20	25	35
5	5.26	32	28	42	56	20	30	40
6	6.9	46	27	40	51	15	30	40
7	6.25	62	33	39	53	20	30	40
8	7.15	82	26	36	46	20	40	60
9	8.2	101	23	34	44	20	45	60
10	8.20	118	23	33	43	0	0	0
Total			247	343	441	150	250	350
2023								
1	4.29	1	22	25	37	0	0	0
2	5.5	7	23	26	33	15	15	35
3	5.13	15	22	30	35	20	20	30
4	5.21	23	25	30	38	20	20	40
5	6.5	38	22	43	55	20	35	45
6	6.19	52	22	41	51	15	32	40
7	7.3	66	33	35	53	20	35	40
8	7.19	82	25	33	44	20	43	65
9	8.6	100	22	35	45	20	50	55
10	8.23	117	20	36	46	0	0	0
Total			236	334	437	150	250	350

### 2.3 Observation items

#### 2.3.1 Meteorological data

A davis wireless vantage pro 2 meteorological station (Davis Instruments, Hayward, USA) is installed in the experimental area to record key meteorological data during the crop growth period. It automatically captures data every 15 minutes, including relative humidity, wind speed, wind direction, air pressure, maximum and minimum temperatures, and rainfall.

#### 2.3.2 *ET_0_* calculation

The FPM method is used to calculate the ET0 of Suaeda salsa during the growth periods of 2021 and 2022, as shown in **Formula (1)**


(1) $$ET_{0}=\frac{0.408\text{Δ}(R_{\text{n}}-G)+\gamma \frac{900}{T_{\text{mean}}+273}u_{2}(e_{s}-e_{a})}{\text{Δ}+\gamma (1+0.34u_{2})}$$

where *R_n_* is the net radiation (MJ·m^−2^·d), *G* is the soil heat flux (MJ·m^−2^·d), *e_s_* is the saturated water vapor pressure of the air (kPa), *Δ* is the slope of the curve between saturated water vapor pressure and temperature (kPa·°C^−1^), *T_mean_* is the daily average temperature (°C), *u_2_* is the wind speed at a height of 2 m above the ground (m·s^−1^), and *e_a_* is the actual water vapor pressure of the air (kPa).

#### 2.3.3 Soil moisture and salinity

During the 2-year experiment, soil samples were collected from each treatment plot 24 hours before and 48 hours after irrigation, every 10 cm from the drip irrigation belt at 0, 10, 20, 30, and 40 cm until a depth of 80 cm. In a portion of collected soil samples, moisture content was measured using the drying method, whereas in the remaining samples, salt content was measured using the residue method. The average values represented the average value of the treatment during the growth period.

#### 2.3.4 Soil matrix suction

Following the completion of the reproductive stage in 2022-2023, undisturbed soil samples were collected from the 0–40 cm soil layer, the moisture contents of which were measured using a 1500F1 bar pressure plate extractor. A soil moisture characteristic curve was subsequently fitted using soil matrix suction as shown in **Formula (2)** (Li et al., 2020):


(2) $$S=623695.54{\text{e}}^{-58.8961\theta }$$

In this formula, S represents the soil matrix suction, kPa, and θ represents the soil moisture content by weight, %.

#### 2.3.5 Distribution of the *S. salsa* root system

**Figure 2:**
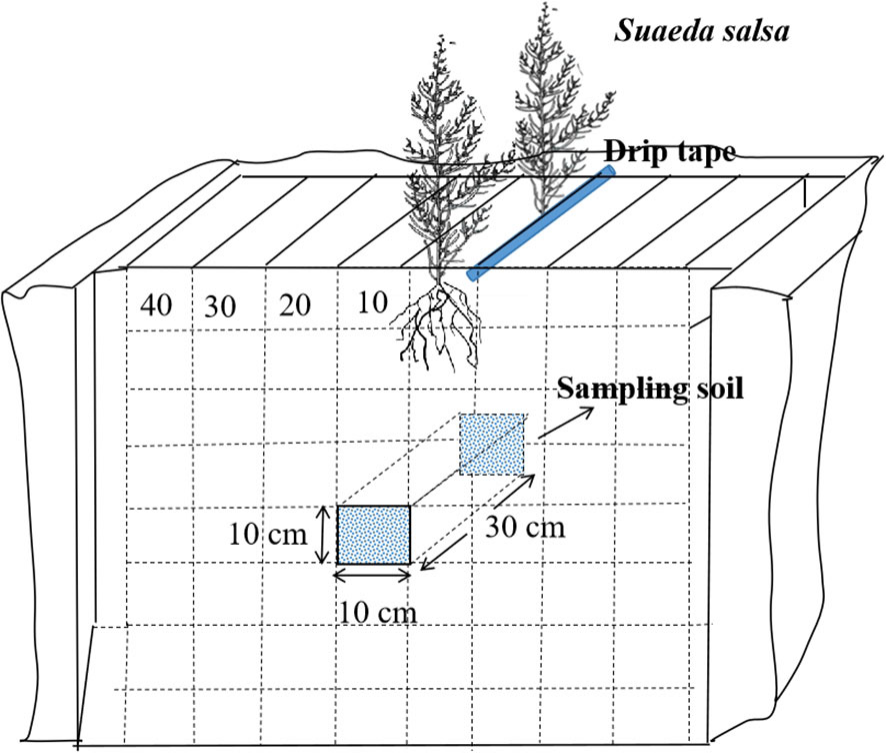
Schematic diagram of root sampling.


During the flowering period of *S. salsa* (August 30, 2022, and August 28, 2023), we analyzed the distribution of the saline–alkali marsh root system by using the diagnostic specimen method. This involved dividing the cotton root layer into multiple grids, each measuring 10 × 10 × 10 cm. Within each grid, both soil and root system were extracted to measure their dry weight and assess the distribution of *S. salsa* root system in the soil. For the sampling process, 4 plants were excavated at a time (2 from each row) located on both sides of the drip irrigation pipe. The excavation extended 30 cm horizontally along the drip irrigation line and vertically to the midpoint between two wide rows. The width of the excavation was 40 cm, and the depth was set at 80 cm, including a 10 cm layer beneath the surface (Li, 2006). **Figure 2** illustrates how this excavation zone was laid out. Root density was calculated using the following **Formula (3)**:

(3) $$RWD=R_{G}/D_{V}$$

In this formula, *RWD* represents the root mass density, (g.cm^-3^); *R_G_* represents the root biomass of different soil layers, (g); *D_v_* represents the volume of different soil layers, (cm^-3^).

#### 2.3.6 Biomass

After harvesting, random *S. salsa* plant samples (1 m^2^ per plot) were collected and separated into seeds, leaves, stems, and roots. These were blanched at 105°C for 10 minutes and then dried at 80°C to constant weight for dry weight measurement.

#### 2.3.7 Crown-to-root ratio

This ratio was calculated using the following **Formula (4)**:


(4) $$C\text{rown}/R\text{oot}=(D_{G}+D_{L}+D_{S})/D_{R}$$

Where *D_G_*, *D_L_*, *D_S_*, and *D_R_* represent the biomass of seeds, leaves, stems, and roots, respectively (t·hm^−2^).

#### 2.3.8 Ash content of *S. salsa*

The ash content was measured based on Chinese national standard GB/T 64382007.Weigh 5.00 g of dried plant material onto a calcined plate, place the calcined plate containing the sample on the electric heating plate and heat it carefully until the sample is carbonized, transfer it to a muffle furnace pre heated to 550 °C and burn for 3 hours, observe if there are carbon particles. If there are no carbon particles, continue to burn in the muffle furnace for 1 hour. If there are carbon particles, cool the calcination plate and wet it with distilled water. Evaporate it in a drying oven at 103 ± 2 °C until dry, and then place the calcination plate in the muffle furnace for 1 hour, cool to room temperature and take it out of the dryer for rapid weighing.

#### 2.3.9 Salt absorption capacity of *S. salsa*

The salt absorption capacity of *S. salsa* was determined usingthe following **Formula (5)** (Wang et al., 2022):


(5) $$S_{P}=D_{G}\times A_{G}+D_{L}\times A_{L}+D_{S}\times A_{S}+D_{R}\times A_{R}$$

In this formula, *S_P_* represents the amount of salt absorbed by *S. salsa* (kg·hm^−2^); *D_G_*, *D_L_*, *D_S_*, and *D_R_* represent the biomass of seeds, leaves, stems, and roots, respectively (kg·hm^−2^); and *A_G_*, *A_L_*, *A_S_*, and *A_R_* represent the ash content of *S. salsa* seeds, leaves, stems, and roots, respectively (g·kg^−1^).

### 2.4 Data processing

Figures were produced using Microsoft Excel 2016. Variance analysis was performed using the SPSS software (version 15.0). All data were analyzed by two-way ANOVA, the normality of residuals and the homogeneity of variance should be tested using two-way ANOVA. The significance was determined at the 5% significance level. All results are presented as means plus or minus the standard error (SE). Graphical representations and illustrations were created using Origin 2020 software(Origin Lab, USA).

## 3 Results

### 3.1 Effect of water–nitrogen coupling on the distribution of soil matrix suction

**Figure 3:**
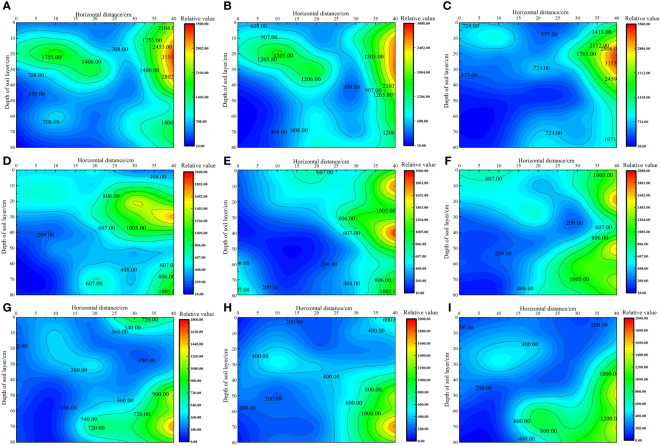
Effect of water–nitrogen coupling on the distribution of soil matrix suction in the *S. salsa* root zone **(A–I)** represent the soil matrix suction of *S. salsa* root by W1N1, W1N2, W1N3, W2N1, W2N2, W2N3, W3N1, W3N2 and W3N3, respectively.

As shown in **Figure 3**, under the same irrigation level, the distribution trend of soil matrix suction across different nitrogen application rates was similar. As irrigation volume increased, the soil’s wetting zone gradually expanded outward and the shape of the wetting zone changed from a “narrow and deep” to a “wide and shallow” pattern. This change was more pronounced in the horizontal direction than in the vertical direction, with soil matrix suction consistently following the order W1 > W2 > W3 at the same location. Under the W1 irrigation level, the lowest soil matrix suction was recorded at the 0 cm level, with average values in the 0–80 cm soil layer ranging from 330.49 to 421.55 kPa. By contrast, the highest soil matrix suction was noted at the 40 cm level, with average values in the 0–80 cm soil layer ranging from 1925 to 2205.54 kPa. Under the W2 irrigation level, the lowest soil matrix suction was recorded at the 0 cm level, with average values in the 0–80 cm soil layer ranging from 252.50 to 292.15 kPa. By contrast, the highest soil matrix suction was noted at the 40 cm level, with average values in the 0–80 cm soil layer ranging from 831.49 to 1159.42 kPa. Under the W3 irrigation level, the lowest soil matrix suction was recorded at the 0 cm level, with average values in the 0–80 cm soil layer ranging from 96.22 to 153.75 kPa. By contrast, the highest soil matrix suction was recorded at the 40 cm level, with average values in the 0–80 cm soil layer ranging from 796.58 to 995.66 kPa.

In previous research, the optimal lower limit of soil field capacity for the growth of *S. salsa* was identified to be 70%. Accordingly, the minimum soil matrix suction corresponding to this soil field capacity was found to be 586.09 kPa. By using this threshold as a benchmark for evaluating the soil wetting area, we determined that most of the soil wetting areas under W2 and W3 irrigation levels were within the range of available water content in soil. At the W2 level, the soil wetting area in the horizontal direction of 0–30 cm and the vertical direction of 0–80 cm were within the range of the available soil water content, and the roots of *S. salsa* were mainly concentrated in this area. At the W3 level, the soil wetting area in the horizontal direction of 0–40 cm and the vertical direction of 0–50 cm were within the range of the available soil water content, and the roots of *S. salsa* were mainly concentrated in this area. However, at the W1 level, which indicated drought stress conditions, the soil matrix potential below the vertical 40 cm layer was low, posing no threat to the water absorption of *S. salsa* roots and allowing the roots to grow deeper into the soil.

### 3.2 Effect of water–nitrogen coupling on soil salt distribution in the *S. salsa* root zone

**Figure 4:**
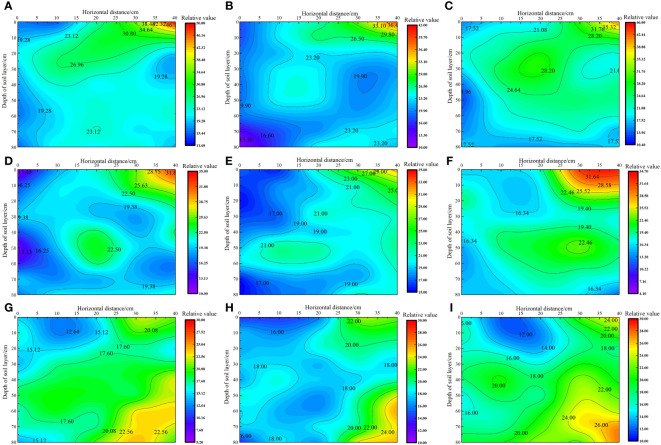
Effect of water–nitrogen coupling on soil salt distribution in the *S. salsa* root zone **(A–I)** represent the soil salt distribution of *S. salsa* root by W1N1, W1N2, W1N3, W2N1, W2N2, W2N3, W3N1, W3N2 and W3N3, respectively.

As shown in **Figure 4**, under the same irrigation level, the distribution trend of soil salt across different nitrogen application rates was similar. As the irrigation volume increased, the soil moisture zone gradually expanded, and the salt content gradually increased from 0 to 40 cm horizontally. The soil salt content followed the order W3< W2< W1 at the same location, with the irrigation volume increasing from W1 to W3 level. The average salt content in each soil layer decreased by 2.84%–26.89% and 3.14%–40.56%, respectively. Under the W1 irrigation level, the lowest salt content in each soil layer was recorded at the 0 cm level, with average values in the 0–80 cm soil layer ranging from 19.02 to 19.745 g·kg^−1^. However, the highest soil salt content was recorded in the 40 cm level, with average values ranging from 24.03 to 25.13 g·kg^−1^ in the 0–80 cm soil layer. In the vertical direction, the average salt content in the soil layer below 40 cm decreased by 6.57% to 38.89% compared with that in the 0–40 cm soil layer. This reduction did not cause stress on the water absorption of the alkali fluffy root system in the saline soil, allowing deeper root growth. Under the W2 irrigation level, the lowest soil salt content was recorded at the 0 cm level, with average values in the 0–80 cm soil layer ranging from 17.17 to 19.23 g·kg^−1^. By contrast, the highest soil salt content was recorded at the 40 cm level, with average values in the 0–80 cm soil layer ranging from 20.29 to 23.14 g·kg^−1^. In the vertical direction, the average salt content in the 0–40 cm soil layer was higher than that in the 40–80 cm soil layer. Under the W3 irrigation level, the lowest soil salt content was recorded at the 0 cm level, with average values in the 0–80 cm soil layer ranging from 16.49 to 17.41 g·kg^−1^. By contrast, the highest soil salt content was recorded at the 40 cm level, with average values in the 0–80 cm soil layer ranging from 20.24 to 22.71 g·kg^−1^. In the vertical direction, the soil salt content in the 0–40 cm soil layer was higher than that in the 40–80 cm soil layer, and the *S. salsa* root system was mainly concentrated in this area.

### 3.3 Effect of water–nitrogen coupling on the root distribution of *S. salsa*

**Figure 5:**
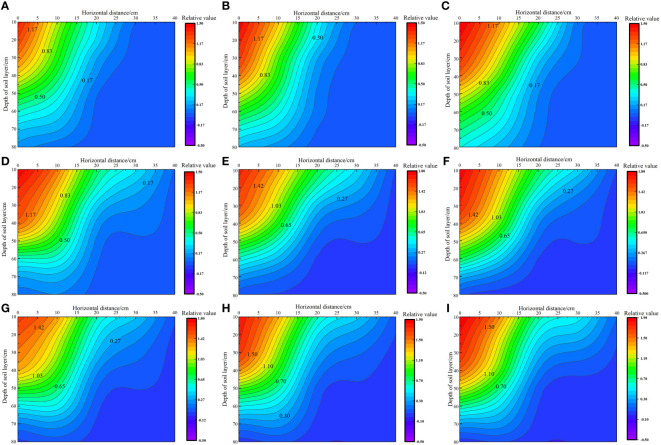
Effect of water–nitrogen coupling on the root distribution of *S. salsa* **(A–I)** represent the root distribution of *S. salsa* root by W1N1, W1N2, W1N3, W2N1, W2N2, W2N3, W3N1, W3N2 and W3N3, respectively.

As shown in **Figure 5**, with the increase in irrigation level and nitrogen application rates, the total root weight density of *S. salsa* in saline soil increased from 17.18 × 10−3 g·cm^−3^ to 27.91 × 10−3 g·cm^−3^. At the same irrigation level, the total root weight density increased by 14.25% to 33.40% when the nitrogen application level increased from N1 to N3. Furthermore, at the same nitrogen application level, the total root weight density increased by 6.51% to 22.75% when the irrigation level increased from W1 to W3. Vertically, 69.44% to 100% of the *S. salsa* root system was distributed in the 0–40 cm soil layer, and the distribution limit depth of the *S. salsa* root system reached 70–80 cm. Horizontally, 55.29% to 58.48% of the root system was distributed in the 0–10 cm soil layer, and the distribution limit depth of the root system reached a maximum of 30–40 cm.

We noted significant differences in root distribution among different water and nitrogen treatments. Under the same irrigation level, with the increase in the fertilizer application rate from N1 to N3, the vertical and horizontal root weight density increased, and the root weight density at 0 cm increased from an average of 0.78 × 10−3 g·cm^−3^ to 1.14 × 10^−3^ g·cm^−3^. Under the same fertilization level, with the increase in irrigation water, the *S. salsa* roots horizontally expanded from 0–20 cm to 0–40 cm. At the W1 irrigation level, only a few roots in the 20–30 cm soil layer expanded to a depth of 30 cm horizontally. At the W2 irrigation level, all roots in the 0–50 cm soil layer expanded to a depth of 30 cm horizontally. At the W3 irrigation level, all roots in the 0–40 cm soil layer expanded to a depth of 40 cm horizontally. In the vertical direction, the limit depth of root distribution remained within 80 cm. With an increase in the irrigation level, the increase in the surface root weight density was significantly greater than that in deep roots. The root weight density in the 0–40 cm layer increased by 43.63%, whereas that in the 40–80 cm layer increased by only 25.87%. In general, with increases in irrigation water and nitrogen application, the root distribution pattern transitioned from a “narrow–deep type” to a “wide–shallow type.”

### 3.4 Effect of water–nitrogen coupling on the biomass and crown–to–root ratio of *S. salsa*

**Figure 6:**
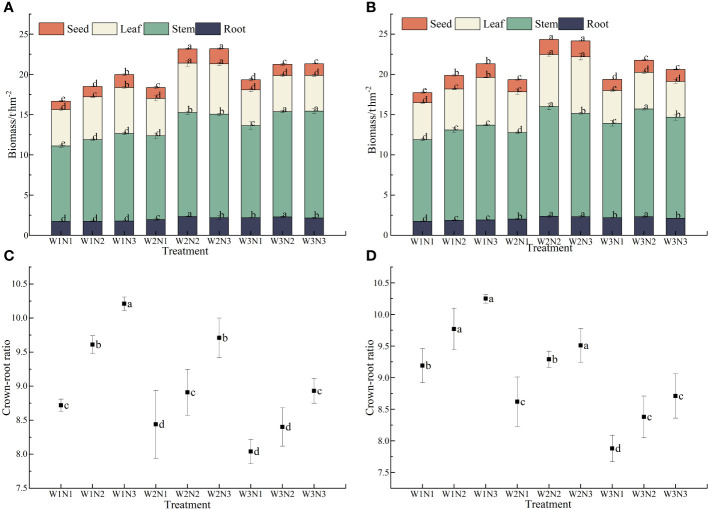
Effects of water and nitrogen coupling on biomass and crown-to-root ratio of *S. salsa* **(A, B)** represent the biomass of different organs of *S. salsa* in 2022 and 2023, respectively. **(C, D)** Represent the crown-to-root ratio of *S. salsa* in 2022 and 2023, respectively. Bars and error bars are the mean±SE (n = 3). Different lowercase letters indicate a significant difference (p < 0.05).

As shown in **Figure 6**, different water and nitrogen treatments significantly affected the biomass of *S. salsa* (*p*< 0.05). At the same irrigation level, the biomass of each organ of *S. salsa* increased with the fertilization level. When the fertilizer level increased from N1 to N3, the biomass of seeds, leaves, stems, and roots increased by 9.43% to 45.61%, by 3.98% to 36.79%, by 9.52% to 25.47%, and by only 1.83% to 18.64%, respectively. At the same fertilization level, the biomass of each organ of *S. salsa* increased initially and then decreased with the increase in irrigation volume (*p*< 0.05). When the irrigation volume increased from W1 to W2, the biomass of seeds, leaves, stems, and roots increased by 15.66% to 25.44%, by 6.46% to 20.90%, by 8.35% to 24.12%, and by only 14.74% to 31.20%, respectively. When irrigation volume increased from W2 to W3, the biomass of seeds, leaves, stems, and roots decreased by 7.34% to 23.18%, by 12.33% to 33.36%, by −9.59% to 0.34%, and by only −9.82% to 4.68, respectively. During the two years, the total biomass of *S. salsa* was the highest in the W2N3 and W2N2 treatments. However, no significant difference was observed between the two treatments, reaching 23.75 t·hm^−2^ and 23.68 t·hm^−2^, respectively. The W1N1 treatment resulted in the lowest total biomass (only 17.21 t·hm^−2^). Water–fertilizer coupling significantly affected the total biomass of *S. salsa* during the 2 years (*p*< 0.05).

Different water and nitrogen treatments significantly affected the crown–to–root ratio of *S. salsa* (*p*< 0.05). Under the same irrigation volume, the crown-to-root ratio of *S. salsa* increased with the fertilization level. When the fertilization level increased from N1 to N3, the crown-to-root ratio increased by 5.40% to 14.24%. Under the same fertilization level, the crown–to–root ratio of *S. salsa* was negatively correlated with irrigation volume. When the irrigation volume increased from W1 to W3, the crown-to-root ratio decreased by 4.75% to 8.22%. The crown-to-root ratio of *S. salsa* was the highest in the W1N3 treatment, reaching 10.21 and 10.25, respectively. The W3N1 treatment resulted in the lowest crown-to-root ratio, reaching only 8.04 and 7.88. Water–fertilizer coupling substantially affected the crown-to-root ratio of *S. salsa* during the 2 years (*p*< 0.05).

### 3.5 Effect of water–nitrogen coupling on the ash content of *S. salsa*

**Figure 7:**
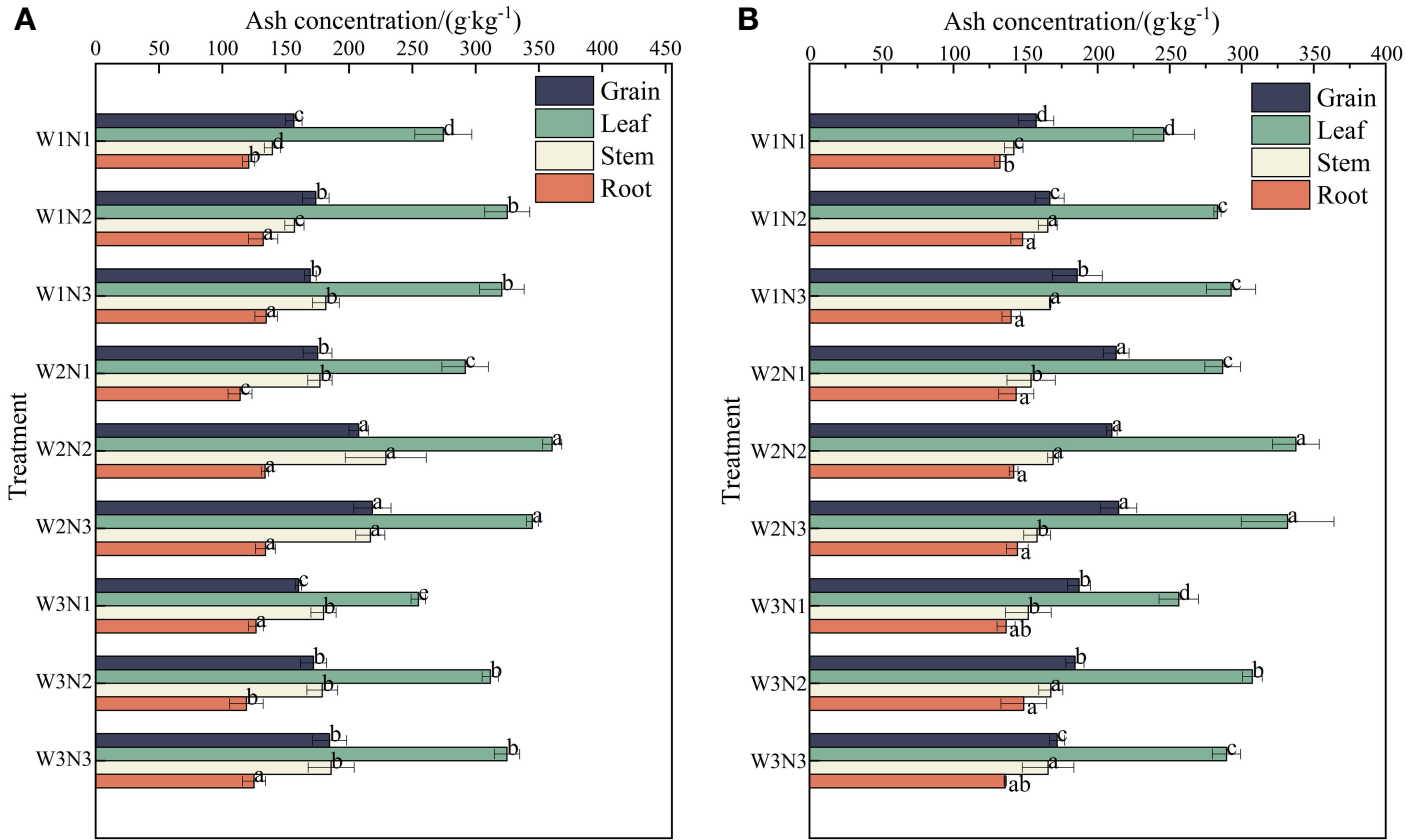
Effect of water–nitrogen coupling on ash content in *S. salsa* **(A, B)** represent the ash content of different organs of *S. salsa* in 2022 and 2023, respectively. Bars and error bars are the mean±SE (n = 3). Different lowercase letters indicate a significant difference (p < 0.05).

As shown in **Figure 7**, during the 2 years, the ash content in the different organs of *S. salsa* varied across different treatments, with the order being leaves > seeds> stems > roots. The ash content was the highest in leaves (255.51 to 349.03 g·kg^−1^), followed by seeds(157.00 to 216.47 g·kg^−1^), stems (140.71 to 199.03 g·kg^−1^), and roots (126.52 to 140.13 g·kg^−1^).

Different water and nitrogen treatments significantly affected the ash content of each organ of *S. salsa* under the same irrigation conditions (*p*< 0.05). The ash content in the leaves, stems, and roots increased first and then decreased with the increase in the nitrogen application level. The ash content increased by 19.55%, 13.08%, and 6.50% from N1 to N2 and decreased by 0.99%, 1.28%, and 1.13% from N2 to N3. Under the same nitrogen application conditions, significant differences were noted between different irrigation amounts. The ash content in the leaves increased first and then decreased with the increase in irrigation volume. The ash content in the leaves. Stems, and roots increased by 12.10%, 10.10%, and 0.48% from W1 to W2 and decreased by 10.75%, 12.75%, and 12.30% from W2 to W3. Under the same irrigation conditions, the ash content in seeds gradually increased with the increase in the nitrogen application level. The average ash content increased by 13.08% from N1 to N2 and by 1.28% from N2 to N3. Under the same nitrogen application conditions, significant differences were observed between different irrigation volumes. The ash content in seeds increased first and then decreased with the increase in irrigation volume. The ash content in seeds increased by 16.18% from W1 to W2 and decreased by 6.30% from W2 to W3.

### 3.6 Effect of water–nitrogen coupling on the salt uptake of *S. salsa*

**Figure 8:**
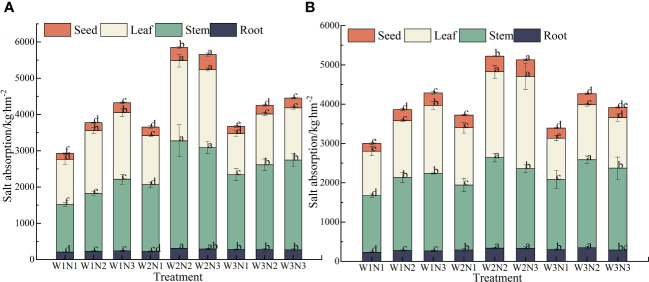
Effect of water–nitrogen coupling on Salt absorption in *S. salsa* **(A, B)** represent the Salt absorption of different organs of *S. salsa* in 2022 and 2023, respectively. Bars and error bars are the mean±SE (n = 3). Different lowercase letters indicate a significant difference (p < 0.05).

As shown in **Figure 8**, during the 2-year experiment, we observed a significant difference in salt uptake among different organs of *S. salsa* across different treatments (*p*< 0.05). The order of salt uptake was stems > leaves> seeds > roots. The salt uptake by the stems, leaves, seeds, and roots ranged from 1374.17 to 2632.59 kg·hm^−2^, from 1191.02 to 2251.28 kg·hm^−2^, from 179.94 to 416.12 kg·hm^−2^, and from 218.84 to 324.48 kg·hm^−2^, respectively.

We noted a significant difference in the total salt uptake of *S. salsa* across different treatments (*p*< 0.05). Under the same irrigation level, the total salt uptake of *S. salsa* increased with the fertilization level. When the fertilization level increased from N1 to N3, the salt uptake increased by 18.38% to 50.11%. Under the same fertilization level, with the increase in irrigation volume, the salt uptake in various organs of *S. salsa* in saline soil first increased and then decreased. When the irrigation volume increased from W1 to W2, the salt uptake in each organ increased by 24.46% to 45.02%. When the irrigation volume increased from W2 to W3, the salt uptake in each organ decreased by 4.20% to 23.08%. During the 2 years, the total salt uptake in the W2N2 treatment was the highest (5536.96 kg·hm^−2^). No significant difference was noted between W2N2 and W2N3 treatments (*p* > 0.05). Correspondingly, the total salt uptake of W1N1 treatment was the lowest (2963.96 kg·hm^−2^). The total salt uptake in the W2N2 treatment was 86.81% higher than that in the W1N1 treatment.

## 4 Discussion

### 4.1 Effect of water–nitrogen coupling on the soil water–salt environment in saline wasteland

Soil matrix suction is substantially affected by irrigation volume, with differences in irrigation levels altering the soil moisture profile and causing spatial changes in soil matrix suction (Liang et al., 2015). The findings of this study revealed that as the irrigation volume increased, the soil wetting zone gradually expanded, with the shape of this zone transitioning from a “narrow deep type” to a “wide shallow type.” The soil matrix suction gradually increased from 0 to 40 cm in the horizontal direction, with its order remaining W1 > W2 > W3 at the same location. Irrigation can maintain optimal water conditions in the soil of the crop root zone, and a higher total soil water potential is beneficial for the absorption of water by crop roots (Guo et al., 2022). However, excessive salt content in saline wastelands can easily lead to an increase in solute suction in the soil of the crop root zone, causing difficulty in water absorption by crops. This not only leads to seed failure or delayed germination but also affects the normal water absorption of crops. In severe cases, it can cause cell shrinkage due to water absorption, resulting in cell death (Feng et al., 2011). In this study, we noted that irrigation water exerted a certain leaching effect on surface soil salt. As the irrigation volume increased, the soil wet zone gradually expanded. The soil solute suction followed the order W3< W2< W1 at the same location, and it decreased by 2.84% to 26.89% and 3.14% to 40.56%, respectively, when the irrigation volume was increased from W1 to W3. This finding aligns with those reported by Li et al. (2015a), Li et al. (2015b) who found that increasing the irrigation quota is beneficial for the downward movement of the salt peak in the soil profile and the reduction of soil solute suction.

Halophytes can grow in high-salt environments because the water potential of their cells is lower than that of the high-salt environment, enabling them to absorb water from the high-salt environment for their growth and development (Guo et al., 2016). In this study, we noted that with increases in fertilizer application and irrigation, the yield of *S. salsa* first increased and then decreased. During the 2-year study period, the total biomass was the highest in W2N3 and W2N2 treatments. Under W2 and W3 irrigation levels, soil matrix suction was within the range of the available soil water content, and no drought stress was noted (**Figure 3**). At this time, the soil salt content under the W2 irrigation level was suitable for the growth of *S. salsa*. This is because halophytes require a moderate salt environment for their growth and development. Any concentration above or below this range would impede their growth and even cause damage (Song et al., 2009).

### 4.2 Effect of water–nitrogen coupling on the root distribution of *S. salsa*

The root system of plants is in direct contact with the soil and is responsible for absorbing water and mineral nutrients, thus substantially affecting the growth and development of the parts above the ground (Min et al., 2014). The soil’s water and salt conditions, along with its physical and chemical properties, primarily affect the growth, distribution, and function of the root system. Soil matrix suction, rather than soil moisture content, is an important factor affecting the distribution of the root system (Liu et al., 2020). This study revealed that under the W1 irrigation level, the average salt content in below the 40-cm soil layer ranged from 6.57% to 38.89%, which was lower than that in the 0–40-cm soil layer, creating a favorable condition for water absorption by the *S. salsa* root system and allowing deeper root growth. This finding is consistent with that reported by Min et al. (2014), who indicated that field crops tend to reduce their shallow root systems while expanding their deeper roots as a response to water stress. Under water stress, the biomass and length of the deeper roots increase, whereas those of the shallow roots decrease. Under the W3 irrigation level, the soil salt content decreased by 3.14% to 40.56% and the soil moisture zone in the horizontal direction of 0–40 cm and the vertical direction of 0–40 cm were both within the range of effective soil water content; the distribution of roots in this region was also noted. Li et al. (2015a) reported that the distribution of cotton roots was significantly affected by soil matrix suction. However, in this study, the distribution of *S. salsa* roots was not only affected by soil matrix potential but also significantly affected by solute potential.

The root system is an important organ for crops and is responsible for absorbing water and nutrients from the soil to maintain normal growth. The degree of root development is largely determined by the supply of water and nutrients in the soil during the growth period of crops. Appropriate water and fertilizer application can promote the growth of roots (Wu et al., 2019). With the increase in irrigation volume and nitrogen application rates, the total root weight density of *S. salsa* in saline soil increased from 17.18 × 10^−3^ g·cm^−3^ to 27.91 × 10^−3^ g·cm^−3^, which is consistent with the findings reported by Tao (2013) In addition, with the increase in irrigation volume and nitrogen application rates, the root distribution of *S. salsa* in saline soil changed from “narrow and deep” to “wide and shallow,” aligning with the results reported by Wang et al. (2011) Drip irrigation, a localized soil moistening method, affects the distribution change. The narrow and deep soil moistening zone with a low soil matrix potential at the surface is not conducive to the expansion of crop root surfaces. Conversely, the wide and shallow soil moistening zone with a high soil matrix potential at the surface is conducive to the growth of cotton roots. Gao et al. (2018) indicated that under spatial soil moisture stress, cotton plants maintain their transport function by branching their main roots, resulting in a shallow distribution of the entire root system and a high root distribution density, which are advantageous for effectively absorption of water and nutrients in the soil’s surface layer.

### 4.3 Effect of water–nitrogen coupling on the crown-to-root ratio of *S. salsa*

Plants adapt to varying site conditions by altering the distribution of photosynthetic products among different organs, leading to changes in their root-to-shoot ratios (Xie et al., 2009). Water is a key factor affecting the growth of above-ground parts and the formation of yield. Water stress severely affects crop growth, causing an increase in the root-to-shoot ratio (Liu et al., 2016). Under regulated deficit irrigation, root biomass decreases, but the root-to-shoot ratio decreases and root activity increases, indicating that although water stress inhibits root growth quantitatively, it exerts a compensatory effect on morphology and function (Zhou et al., 2020). This finding is consistent with the results of this study, which indicated that the root-to-shoot ratio of *S. salsa* was negatively correlated with irrigation volume. When the irrigation volume increased from W1 to W3, the root-to-shoot ratio decreased by 4.75% to 8.22%. This finding indicated that *S. salsa* exhibits certain adaptability under drought conditions by increasing the allocation of biomass to the roots (i.e., reducing the root-to-shoot ratio), expanding the surface area of the roots, facilitating the extraction of water from deep soil layers, and maintaining water balance within the system (Wu et al., 2019).

With the increase in the nitrogen fertilizer application rate, the root-to-shoot ratio decreased, and nitrogen fertilizer application considerably promoted the growth of the aboveground parts (Hu et al., 2017). Under the same irrigation volume, the crown-to-root ratio of *S. salsa* increased with the fertilization level. When the fertilization level increased from N1 to N3, the crown-to-root ratio increased by 5.40% to 14.24%. This finding is consistent with that reported by Dong et al (Hu et al., 2017), who indicated that the application of nitrogen fertilizer significantly increased the plant height, plant density, leaf number, and leaf area of *Leymus chinensis*, thereby promoting its nutritional growth and increasing its crown-to-root ratio. Zhang et al. (2007) found that water and nitrogen more substantially affect the aboveground parts of halophytes than the underground parts. This adaptation mechanism is crucial for halophytes to cope with high-salt environments because it rapidly increases the biomass of the aboveground parts, thereby diluting salt concentration (Min et al., 2020). This finding is consistent with the results of this study, which revealed that the effect of water–fertilizer coupling on the crown-to-root ratio of *S. salsa* was significant (p< 0.05) over two years, with the W1N3 treatment resulting in the highest crown-to-root ratios of 10.21 and 10.25, respectively. The W3N1 treatment yielded the lowest crown-to-root ratios of 8.04 and 7.88, respectively.

### 4.4 Effect of water–nitrogen coupling on the salt absorption capacity of *S. salsa*

The ability of halophytes to uptake salt, a crucial factor in assessing their efficacy in improving saline soil, is influenced by both their biomass and ash content (Zhang et al., 2013). Liang et al. (2013) observed that topdressing with nitrogen fertilizer increased the biomass of the stems, seeds, and leaves of S, salsa, thereby increasing its salt uptake. This finding is inconsistent with the results of this study. In this study, an increase in the nitrogen application level significantly increased not only the biomass but also the ash content of each organ. The combined effect of the two factors significantly increased the salt uptake of *S. salsa*. This finding is consistent with that reported by Pan et al. (2014). The aboveground biomass and ash content tended to increase with the increase in the nitrogen application rate. Jiang et al. (2012) reported that increasing nitrogen fertilizer can significantly promote the growth of halophyte roots, enhancing the absorption and utilization of salt ions through well-developed root morphology. Song et al. demonstrated that increasing the accumulation of N in *S.salsa*.*S. salsa* under salt stress is beneficial for improving the nutrient balance of plants under salt stress and facilitating further absorption of Na^+^ (Song et al., 2009, 2016). In addition, different irrigation volumes substantially affect ash content (Pan et al., 2014). Che and Zhao (2000) determined that although the salt ion content in various organs of *S. salsa* increases with external salinity, the relationship is not linearly proportional. This finding is consistent with the results of this study. Our results revealed that the ash content in the leaves, stems, and roots of *S. salsa* in saline soil increases initially and then decreases with the increase in irrigation volume.

Bai et al. (2017) determined that the absorption of nitrate nitrogen by halophytes is closely related to soil moisture and nitrogen application. They found that optimal soil moisture and nitrogen levels can enhance the uptake of nitrate nitrogen. For *S. salsa*, nitrate nitrogen serves not only as a nutrient but also plays a crucial role in osmotic regulation. This dual function not only increases its salt tolerance but also improves its salt absorption capacity (Song et al., 2006; Zhang, 2021). This finding is consistent with the results of this study. Our findings revealed a significant difference in the total salt uptake of *S. salsa* under different water and nitrogen treatments (p< 0.05). During the 2-year study period, the highest total salt uptake was 5536.96 kg·hm^−2^ under the W2N2 treatment.

## 5 Conclusion

Under the same irrigation level, the distribution trend of soil substrate suction was similar across different nitrogen application rates. The majority of the soil moisture zones under W2 and W3 irrigation levels were within the range of effective soil moisture content. Under the W2 irrigation level, the soil salt content was optimal for the growth and development of *S. salsa*, whereas under the W1 irrigation level, the plants experienced drought and salt stress. With the increase in irrigation volume and nitrogen application rates, the total root weight density of the saline alkali fluffy root system increased, and the root distribution changed from the “narrow deep type” to “wide shallow type.” Under the same nitrogen application rate, with the increase in irrigation volume, the biomass, ash content, and salt uptake of *S. salsa* in saline soil first increased and then decreased, reaching the maximum value at the W2 irrigation level. At the same time, no significant differences in biomass, ash content, and salt uptake were observed between N2 and N3 treatments (*p* > 0.05). In summary, the W2N2 treatment is suitable for the drip irrigation of *S. salsa* in extremely arid areas.

## Data availability statement

The raw data supporting the conclusions of this article will be made available by the authors, without undue reservation.

## Author contributions

QX: Data curation, Formal analysis, Writing – original draft. HL: Funding acquisition, Resources, Writing – review & editing. ML: Resources, Supervision, Writing – review & editing. PG: Conceptualization, Visualization, Writing – review & editing. PL: Project administration, Supervision, Writing – review & editing. YX: Methodology, Visualization, Writing – review & editing. QZ: Conceptualization, Supervision, Writing – review & editing. HX: Investigation, Writing – review & editing.
